# Simvastatin Suppresses Human Breast Cancer Cell Invasion by Decreasing the Expression of Pituitary Tumor-Transforming Gene 1

**DOI:** 10.3389/fphar.2020.574068

**Published:** 2020-11-04

**Authors:** Litian Yin, Zhongmei He, Bing Yi, Linyuan Xue, Jianxin Sun

**Affiliations:** ^1^ Center for Translational Medicine, Department of Medicine, Thomas Jefferson University, Philadelphia, PA, United States; ^2^ Key Laboratory for Cellular Physiology, Ministry of Education, Department of Physiology, Shanxi Medical University, Taiyuan, China

**Keywords:** pituitary tumor-transforming gene 1, statins, simvastatin, breast cancer, cell migration

## Abstract

Statins, or 3-hydroxy-3-methylglutaryl-coenzyme A reductase inhibitors, have been widely used to lower cholesterol and prevent cardiovascular diseases. Recent preclinical and clinical studies have shown that statins exert beneficial effects in the management of breast cancer, while the underlying mechanisms remain to be elucidated. Herein, we sought to investigate the effect of statins on the expression of pituitary tumor-transforming gene 1 (PTTG1), a critical gene involved in human breast cancer invasion and metastasis. Our results showed that PTTG1 is highly expressed in malignant Hs578T and MDA-MB-231 breast cancer cell lines as compared with normal or less malignant breast cancer cells. Furthermore, we found that the expression of PTTG1 was markedly suppressed by lipophilic statins, such as simvastatin, fluvastatin, mevastatin, and lovastatin, but not by hydrophilic pravastatin. In a dose and time dependent manner, simvastatin suppressed PTTG1 expression by decreasing PTTG1 mRNA stability in MDA-MB-231 cells. Both siRNA-mediated knockdown of PTTG1 expression and simvastatin treatment markedly inhibited MDA-MB-231 cell invasion, MMP-2 and MMP-9 activity, and the expression of PTTG1 downstream target genes, while ectopic expression of PTTG1 promoted cancer cell invasion, and partly reversed simvastatin-mediated inhibition of cell invasion. Mechanistically, we found that inhibition of PTTG1 expression by simvastatin was reversed by geranylgeranyl pyrophosphate, but not by farnesyl pyrophosphate, suggesting the involvement of geranylgeranyl synthesis in regulating PTTG1 expression. Our results identified statins as novel inhibitors of PTTG1 expression in breast cancer cells and provide mechanistic insights into how simvastatin prevent breast cancer metastasis as observed in recent preclinical and clinical studies.

## Introduction

Breast cancer is by far the most frequent cancer in women (23% of all cancers) and the second-most frequent cancer when both sexes are considered (Tampaki et al., 2017). Invasion of cancer cells into surrounding tissue and the vasculature is an initial step during tumor metastasis and considered as a clinical challenge of breast cancer treatment (Hainaut and Plymoth, 2013; Chou et al., 2016; Butti et al., 2019). Hence, it is of a great significance to seek novel breast cancer therapeutic targets to prevent breast caner metastasis (Esteva et al., 2019).

Statins, 3-hydroxyl-3-methyl glutaryl coenzyme A reductase inhibitors, are widely prescribed drugs for the treatment of high cholesterol and atherosclerotic coronary artery disease by inhibiting the rate-limiting enzyme in the cholesterol biosynthetic pathway, 3-hydroxy-3-methylglutaryl-coenzyme A (HMG-CoA) reductase (Liao, 2005; Zhou and Liao, 2009). Recently, statins have also received attention regarding unpredictable benefits to reduce breast cancer progression and mortality (Liu et al., 2015; Borgquist et al., 2018; Gobel et al., 2019) and restrain breast cancer cell migration (Ginestier et al., 2012; Van Wyhe et al., 2017; Gobel et al., 2019). Particularly, simvastatin, as one of the most commonly used statins, has demonstrated significant beneficial effects in reducing breast cancer metastasis and recurrence (Ahern et al., 2011; Chae et al., 2011; Clendening and Penn, 2012; Beckwitt et al., 2018b; Li et al., 2019). However, the underlying mechanisms by which statins inhibit tumor cell metastasis are not fully understood, although several studies have implicated the Rho/ROCK pathway, which is inhibited by statin treatment, as a key mechanism involved in regulation of cancer survival, proliferation, and invasion by statins (Denoyelle et al., 2001; Demierre et al., 2005).

Pituitary tumor-transforming gene 1 (PTTG1) was initially isolated from rat GH-secreting pituitary cell lines by differential mRNA display (Heaney et al., 1999), and subsequently identified as a vertebrate securin, which regulates sister-chromatid separation (Smith et al., 2010). It has been increasingly realized that PTTG1 plays critical roles in regulating cell replication, DNA damage/repair, organ development, and metabolism (Vlotides et al., 2007; Quereda and Malumbres, 2009). Importantly, PTTG has been identified as a key signature gene implicated in tumor metastasis (Tanase et al., 2009; Tong and Eigler, 2009; Liao et al., 2012), and its expression is significantly unregulated in various tumors including pituitary, thyroid, colon, ovary, testis, lung, and breast (Vlotides et al., 2007; Espina et al., 2009; Chen et al., 2011; Liu et al., 2015). Furthermore, overexpression of PTTG1 has been reported to enhance cell proliferation, induce cellular transformation, and promote *in vivo* tumor formation (Vlotides et al., 2007; Yoon et al., 2012). PTTG1 has been shown to be localized in both cytoplasmic and nuclear fractions, and function as a transcriptional activator and securing protein (Quereda and Malumbres, 2009). Indeed, PTTG1 has been shown to transcriptionally activate expression of a wide range of target genes, including c-Myc, FGF-2, cyclin D3, p21, and MMP-2, most of which are critically involved in cancer proliferation and metastasis (Espina et al., 2009; Tong and Eigler, 2009; Reeves et al., 2012). Since PTTG1 is highly expressed in a variety of cancers, understanding the mechanism underlying regulation of PTTG1 expression is essential for developing novel therapeutic strategies.

In the present study, we found that statins potently inhibit PTTG1 expression in breast cancer cells, which led to a marked inhibition of cancer cell invasion. Mechanistically, we demonstrated that simvastatin inhibits PTTG1 expression through decreasing PTTG1 mRNA stability in breast cancer cells.

## Materials and Methods

### Reagents

Simvastatin (purity ≥97%), mevalonate (Me) (purity ≥96%), geranylgeranyl pyrophosphate (GGPP) (purity ≥95%) and farnesyl pyrophosphate (FPP) (purity ≥95%) were obtained from Sigma-Aldrich (Sigma, USA). The concentration of simvastatin was chosen based on previous experiments (Gopalan et al., 2013; Zongping et al., 2015). ROCK inhibitor Y27632 (purity ≥98%) was obtained from Enzo Life Sciences (Enzo Life Sciences, NY, USA). Lovastatin (purity ≥99%) and mevastatin (purity ≥99%) were obtained from Selleck (Selleck, Houston, TX, USA), fluvastatin (purity ≥98%) and pravastatin ((purity ≥99%) were obtained from MedChemExpress (MCE, NJ, USA).

### Cell Culture

The breast cancer cell line MDA-MB-231 (ATCC, Manassas, VA, USA) was maintained in DMEM medium (Corning, Gaithersburg, MD, USA) supplemented with 10% heat-inactivated fetal bovine serum (FBS) (Gibco, Gaithersburg, MD, USA), 100 U/ml of penicillin G sodium, and 100 μg/ml streptomycin sulfate (Invitrogen, Grand Island, NY, USA). Hs578T (ATCC; Manassas, VA, USA) were maintained in DMEM medium supplemented with 2 mM glutamine, 10 μg/ml bovine insulin, 10% FBS, 100 U/ml of penicillin G sodium, and 100 μg/ml streptomycin sulfate. SK-BR-3 (ATCC; Manassas, VA, USA) were maintained in McCoy’s 5A medium (Thermo Fisher Scientific, MA, USA) supplemented with 10% FBS. MCF7 (ATCC; Manassas, VA, USA) were maintained in DMEM medium, supplemented with 0.01 mg/ml human recombinant insulin, 10% FBS. MCF-10A cells (ATCC, Manassas, VA, USA) were cultured in Mammary Epithelial Cell Growth Medium (MECG) BulletKit (Lonza).

### Plasmid Construction

Plasmid construction was performed as described previously (Qin et al., 2014). The human PTTG1 gene (CAG33416.1, 202 amino acids) was PCR amplified from human cDNA templates. To construct mammalian expression vector bearing Flag tagged PTTG1, the PTTG1 coding sequence was amplified by sense primer 5′-GAGA GAA TTC A ATG GCT ACT CTG ATC TAT G-3′ and anti-sense primer 5′-GAGA GGA TCC CAC ACA AAC TCT GAA GCA CT-3′ and then subcloned into the *Eco*RI and *Bam*HI sites of the pCMV2-Flag vector.

### Small Interfering RNA Transfection

Human PTTG1 siRNA (Sigma, SASI-Hs02-00337129) and negative control siRNA (Mission siRNA Universal Negative Control) (Sigma-Aldrich, St. Louis, MO, USA) were transfected into MDA-MB-231 cells with Lipofectamine RNAiMAX transfection Reagent (Invitrogen, Grand Island, NY, USA) in OPTI-MEM (Thermo Fisher Scientific, Grand Island, NY, USA) according to the manufacturer’s recommendation. 4 h after transfection, the medium was replaced by DMEM medium supplemented with 10% FBS and cultured for an additional 48 h, and then treated with vehicle or various concentrations of statins as indicated, cells were then harvested for protein or RNA extraction to assess expression of the target genes.

### Real-Time PCR

Total RNAs were extracted from transfected cells by TRIzol (Life Technologies) using the manufacturer’s protocol and reconstituted in 1.0 μg/μL with nuclease-free water. For quantitative reverse transcription-PCR, cDNA was synthesized from total RNA using Olig-dT primer. qRT-PCR primers used for amplification of targeted genes were: human PTTG1 (forward primer: 5′-CCA GAA TGG CTA CTC TGA TCT ATG-3′, reverse primer: 5′-CAC ACA AAC TCT GAA GCA CTA AG-3′); human c-Myc (forward primer: 5′-CCT​GGT​GCT​CCA​TGA​GGA​GAC-3′, reverse primer: CAG​ACT​CTG​ACC​TTT​TGC​CAG​G); human FGF-2 (forward primer: 5′-AGC​GAC​CCT​CAC​ATC​AAG-3′, reverse primer: 5′-ATC​TTC​CAT​CTT​CCT​TCA​TAG​C-3′); human cyclin D3 (forward: 5′-TGC​CAC​AGA​TGT​GAA​GTT​CAT​T-3′, reverse primer: 5′-CAG​TCC​GGG​TCA​CAC​TTG​AT-3′); human p21 (forward primer: 5′-GAC​ACC​ACT​GGA​GGG​TGA​CT-3′, reverse primer: 5′-CAG​GTC​CAC​ATG​GTC​TTC​CT-3′); human 18s (forward primer: 5′-GTA ACC CGT TGA ACC CCA TT-3′, reverse primer: 5′-CCA TCC AAT CGG TAG TAGCG-3′).

### Western Blotting

Cellular proteins were extracted in RIPA buffer (25 mM Tris-HCl, pH 7.6, 150 mM NaCl, 1% Nonidet P-40, 1% sodium deoxycholate, 0.1% SDS) supplement with proteinase inhibitor mixture containing 2 mM phenylmethylsulfonyl fluoride (PMSF), 20 μg/ml aprotinin, 10 μg/ml leupeptin. Cell lysates were rocking 1 h at 4°C, then centrifuged at 14,000 rpm at 4°C for 20 min. Cell lysates were then subjected to SDS-PAGE and transferred to a nitrocellulose membrane (Bio-Rad Laboratories, Hercules, CA, USA). Blots were blocked with 5% non-fat milk in PBS and then incubate with diluted anti-PTTG1 antibody (Santa Cruz, 56207, 1:1,000), anti-Flag antibody (Genescript, A00187, 1:1,000), and anti-GAPDH antibody (Santa Cruz, 32233, 1:1,000). Blots were visualized by either IRDye 700 or 800 labeled secondary antibodies (1:10,000, LICOR, 926-32212, 926-68073 and 925-68074) and then were visualized on an Odyssey Imaging System (Licor). The intensity of the bands was quantified by the Odyssey software (Li-Cor Biosciences, Lincoln, NE, USA).

### Matrigel Invasion Assay

At 24 h after transfection, cells were collected and suspended in serum-free medium. Cells (4 × 10^4^) in 0.2 ml serum-free medium were plated in the top chamber with a Matrigel-coated membrane (24-well insert, pore size, 8 μm, Corning, USA), with 10% FBS as an attractant (Di et al., 2015). The cells were then incubated for 48 h. The cells that did not invade through the pores were removed, and the filter was stained with crystal violet for visualization and counting.

### Gelatin Zymography

Measurement of matrix metalloproteinase-2 (MMP-2) and -9 (MMP-9) activity by gelatin zymography was performed as described previously (Sun and Hemler, 2001). MDA-MB-231 cells were seeded in 1,000 μL of DMEM with 10% fetal bovine serum in a 6 well plate. Cells were transfected with Lipofectamine RNAiMAX transfection Reagent (Invitrogen, Grand Island, NY, USA) according to the manufacturer’s protocol. 72 h after transfection, cells were washed with PBS, and cultured in DMEM medium. After 16–24 h incubation, the medium was harvested and cleared by centrifugation at 12,000 rpm for 10 min and subjected to zymography under non-denaturing conditions using 8.0% SDS-polyacrylamide gels containing 1 mg/ml gelatin. The gels were incubated at 37°C overnight in 50 mM Tris (pH 7.5), 5 mM CaCl_2_, and 1 mM ZnCl_2_, and then stained with Coomassie Brilliant Blue R250. Destained gel images were captured by Odyssey Imaging System (Li-Cor Biosciences, Lincoln, NE, USA). ImageJ (NIH) was used to quantify zymographic band intensities.

### Determination of Message RNA Stability

Actinomycin D (5 μg/ml), an mRNA synthesis inhibitor, was added to cells under simvastatin experimental conditions (Yang et al., 2016). Total RNA was extracted at 0, 3, 6, 12, 18, and 24 h after the addition of actinomycin D, and qRT-PCR was then performed. mRNA decay curves were constructed and the half-life (t_1/2_) was calculated from the curves as we described recently (Yan et al., 2008).

### Statistical Analysis

All values are expressed as the mean ± S.E. Comparisons between two groups were analyzed by *t* test while comparisons between more than two groups were made using one-way ANOVA followed by the Tukey’s post-test. *p* < 0.05 was considered to indicate a statistically significant result. All statistical analyses were performed via GraphPad Prism7.

## Results

### Statins Inhibit Expression of Pituitary Tumor-Transforming Gene 1 in Breast Cancer Cells

**FIGURE 1:**
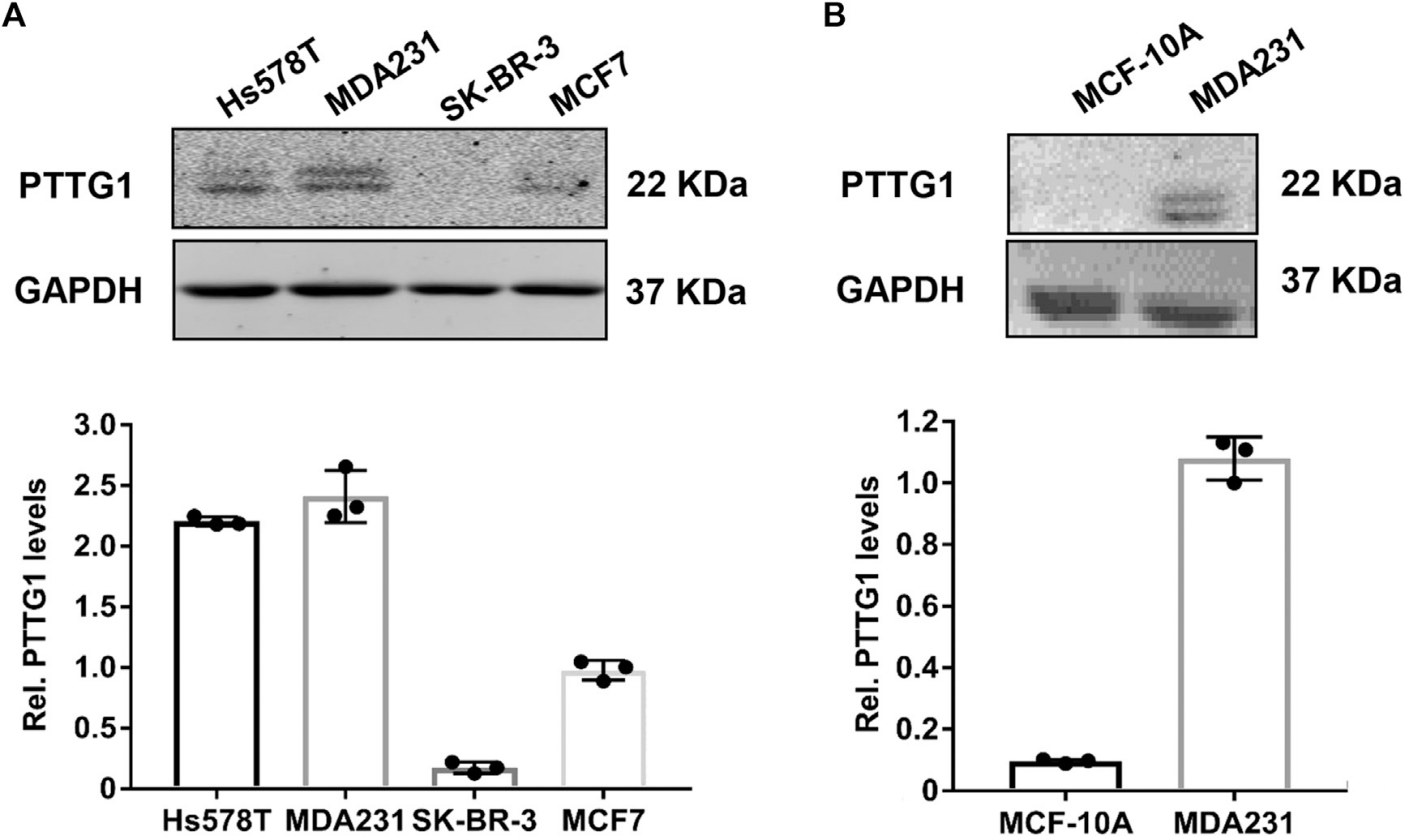
Expression of PTTG1 in human breast cancer cell lines. Expression of PTTG1 in human breast cancer cells **(A)** and normal breast epithelial MCF-10A cells **(B)** was determined by western blot. 30 μg of total cellular lysates obtained from each of the indicated cell lines were subjected to western blot analysis using a PTTG1 antibody.

**FIGURE 2:**
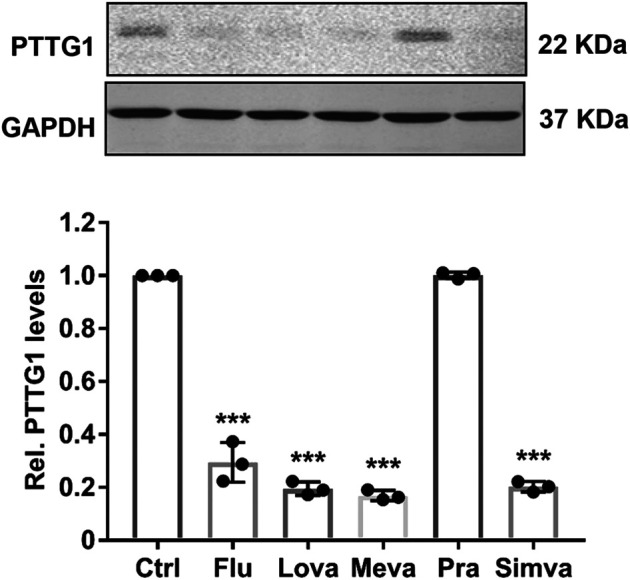
Effect of statins on the expression of PTTG1 in MDA-MB-231 cells. MDA-MB-231 cells were treatment with either control vehicle (Ctrl) or 5 μmol/L of fluvastatin (Flu), lovastatin (Lova), mevastatin (Meva), pravastatin (Pra), and simvastatin (Simva). 24 h after treatment, the expression of PTTG1 was determined by western blot (*n* = 3). ***p < 0.001 vs. control.

To substantiate the functional significance of PTTG1 in breast cancer malignancy, we determined the expression of PTTG1 in four types of different breast cancer cells, with different metastatic capacities. Consistent with previous report (Yoon et al., 2012), we showed that the expression of PTTG1 is significantly higher in malignant Hs578T and MDA-MB-231 breast cancer cell lines than that in less malignant SK-BR3 and MCF-7 breast cancer cells and normal breast epithelia cells, as determined by western blot (Figure 1), indicating the involvement of PTTG1 in breast cancer malignancy. Furthermore, we determined the effects of statins on PTTG1 expression in MDA-MB-231 breast cancer cell line, which expresses a high level of endogenous PTTG1. In this regard, MDA-MB-231 cells were treated with four lipophilic statins (simvastatin, fluvastatin, mevastatin, and lovastatin) and one hydrophilic statin (pravastatin) at a concentration of 5 μmol/L for 24 h, PTTG1 expression was then determined by western blot. As shown in Figure 2, expression of PTTG1 was markedly inhibited by all four lipophilic statins, including fluvastatin, lovastatin, mevastatin, and simvastatin, but not by hydrophilic pravastatin. On a molar basis, we found that all four lipophilic statins exhibit an equal potency. These results suggest that lipophilic and hydrophilic statins may exert differential effects on PTTG1 expression in MDA-MB-231 cells.

### Simvastatin Attenuates Pituitary Tumor-Transforming Gene 1 Expression in MDA-MB-231

**FIGURE 3:**
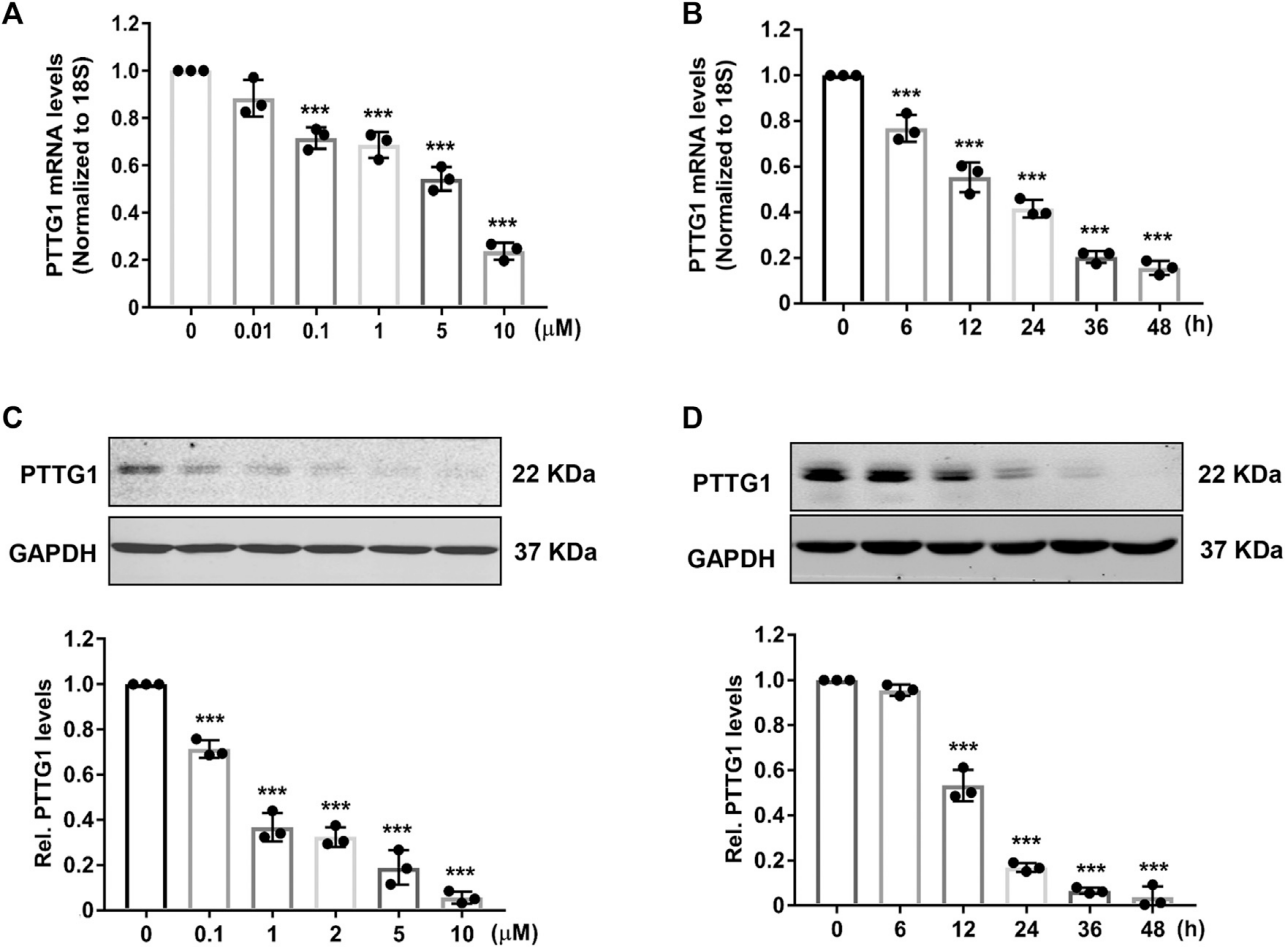
Simvastatin inhibits PTTG1 expression in MDA-MB-231 cells. **(A)** MDA-MB-231 cells were treated with different doses of simvastatin, 24 h after treatment, expression of PTTG1 mRNA was determined by qRT-PCR (*n* = 3). ***p < 0.001 vs. treatment with vehicle. **(B)** MDA-MB-231 cells were treated with 5 μmol/L simvastatin for different time points as indicated, the expression of PTTG1 was determined by qRT-PCR (*n* = 3). ***p < 0.001 vs. time at 0 h. **(C)** MDA-MB-231 cells were treatment with different concentrations of simvastatin for 24 h, the levels of PTTG1 protein was then determined by western blot (*n* = 3). ***p < 0.001 vs. treatment with vehicle. **(D)** MDA-MB-231 cells were treatment with 5 μmol/L simvastatin for different time points as indicated, the levels of PTTG1 protein was determined by western blot (*n* = 3). ***p < 0.001 vs. time at 0 h.

Among different statins, simvastatin has been shown to demonstrate better outcomes in patients with breast cancer (Ahern et al., 2011), thus it was chosen to investigate the dose and time dependent effects of statins on PTTG1 expression in MDA-MB-231 cells. As shown in Figure 3, we found that simvastatin markedly inhibited the expression of PTTG1 in a dose and time-dependent manner with an IC_50_ of approximately 0.85 μmol/L, as determined by both real-time PCR and western blot analysis. Together, these results demonstrated a potent inhibitory effect of simvastatin on PTTG1 expression in MDA-MB-231 cells.

### Statin-Induced Inhibition of Pituitary Tumor-Transforming Gene 1 Is Reversed by Mevalonate and GGPP, but Not by Farnesyl Pyrophosphate

**FIGURE 4:**
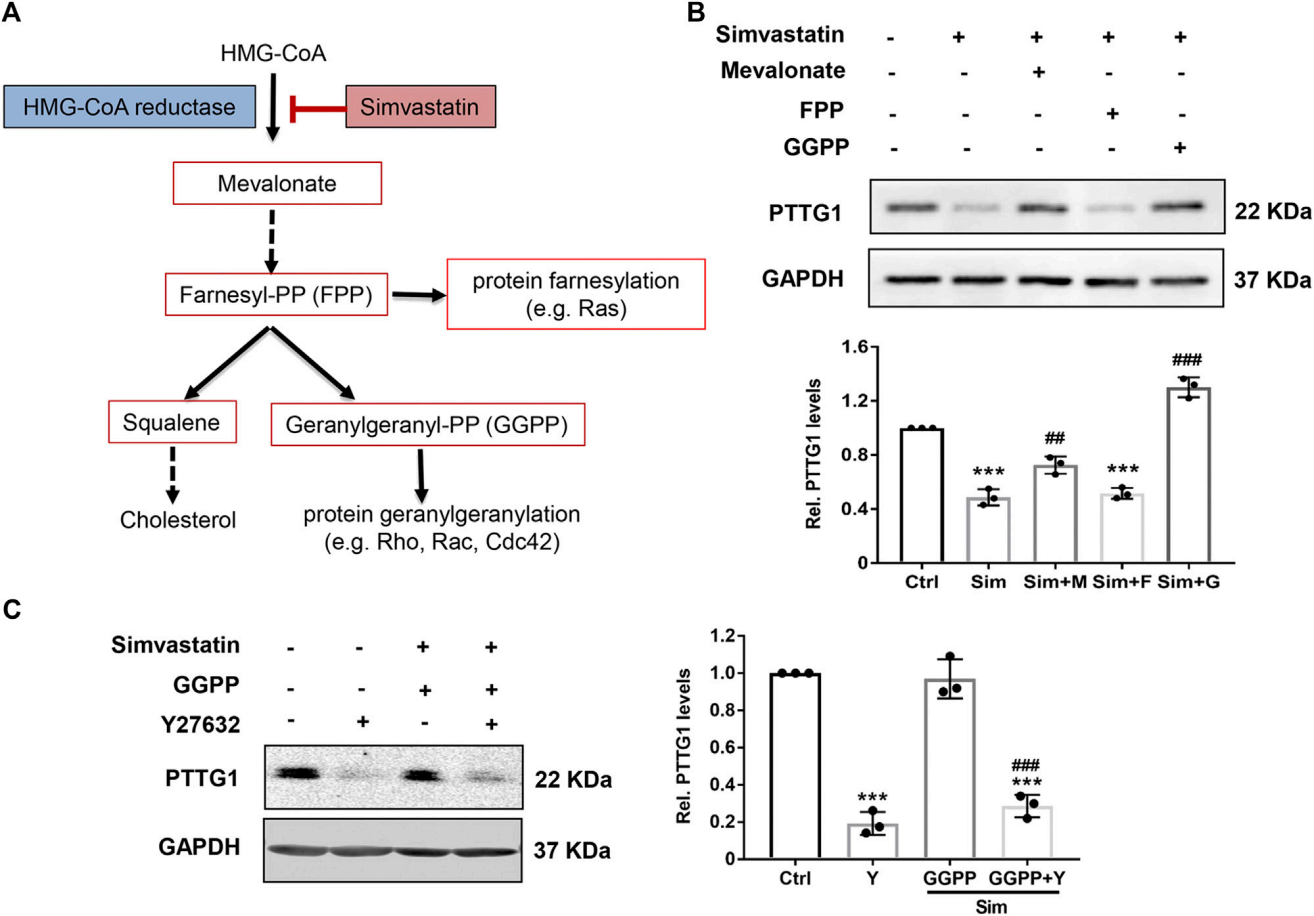
Simvastatin induced inhibition on PTTG1 is reversed by mevalonate and GGPP, but not FPP. **(A)** Schematic overview of the mevalonate pathway. **(B)** Statin-induced inhibition on PTTG1 expression was reversed by mevalonate and GGPP, but not FPP. MDA-MB-231 cells were incubated for 24 h in the presence of control vehicle, 5 μmol/L simvastatin (Sim), 5 μmol/L simvastatin (Sim) + 100 μmol/L mevalonate (M), 5 μmol/L simvastatin (Sim) + 10 μmol/L GGPP (G), or 5 μmol/L simvastatin (Sim) + 10 μmol/L FPP (F). Cells were then harvested and cellular lysates were processed for western blot. Data are presented as mean ± SEM (*n* = 3). ***p < 0.001 vs. control, ^##^ *p* < 0.01 vs. cells treated with simvastatin alone. **(C)** Effects of Y27632 on simvastatin induced inhibition of PTTG1 expression. MDA-MB-231 cells were treated with either vehicle, Y27632 (Y, 10 μmol/L), simvastatin (Sim, 5 μmol/L) + GGPP (10 μmol/L), or simvastatin (5 μmol/L) + GGPP (10 μmol/L) + Y27632 (10 μmol/L) for 24 h. Expression of PTTG1 was then determined by western blot. ***p < 0.001 vs. Ctrl, ^###^ *p* < 0.001 vs. cells treated with simvastatin/GGPP.

In addition to inhibiting l-mevalonate synthesis, HMG-CoA reductase inhibitors also prevent the synthesis of other important isoprenoid intermediates of the cholesterol biosynthetic pathway, such as farnesyl pyrophosphate (FPP) and geranylgeranyl pyrophosphate (GGPP) (Figure 4A), which are the isoprenoids important for the post-translational modification of variety of signaling proteins, including the *γ* subunit of heterotrimeric G proteins, Ras, and Ras-like proteins, such as Rho, Rab, Rac, Ral, or Rap (Laufs and Liao, 1998; Hashemi et al., 2017; Fatehi Hassanabad, 2019). The role that isoprenoids play in regulating PTTG1 expression, however, is not known. To determine which downstream isoprenoid intermediate in the cholesterol biosynthetic pathway regulates PTTG1 expression, we treated MDA-MB-231 cells in the presence of either mevalonate, FPP or GGPP. As shown in Figure 4B, simvastatin-mediated inhibition of PTTG1 expression was completely reversed by co-treatment of either mevalonate or GGPP, but not by FPP. These findings indicate that PTTG1 expression is positively regulated by geranylgeranyl synthesis.

The geranylgeranylation of the small GTPases such as RhoA and RhoB is essential for their membrane translocation from the cytosol (Rikitake and Liao, 2005; Hashemi et al., 2017). To determine whether the inhibition of Rho could attenuate PTTG1 expression, MDA-MB-231 cells were treated with simvastatin in the presence and absence of a potent and specific Rho associated protein kinase (ROCK) inhibitor Y27632 and GGPP. As shown in Figure 4C, similar to the effect of simvastatin, treatment with Y27632 led to a marked inhibition on PTTG1 expression. Furthermore, in the presence of Y27632, restoration of simvastatin-mediated inhibition on PTTG1 expression by GGPP was completely abolished. Together, these data suggest that simvastatin attenuates the expression PTTG1 via inhibiting protein geranylgeranylation. It would be interesting to further examine whether simvastatin inhibits PTTG1 expression through acting on the Rho/ROCK pathway. Some studies are ongoing.

### Simvastatin Inhibits Pituitary Tumor-Transforming Gene 1 Expression Through Destabilizing Pituitary Tumor-Transforming Gene 1 mRNA Half-Life

**FIGURE 5:**
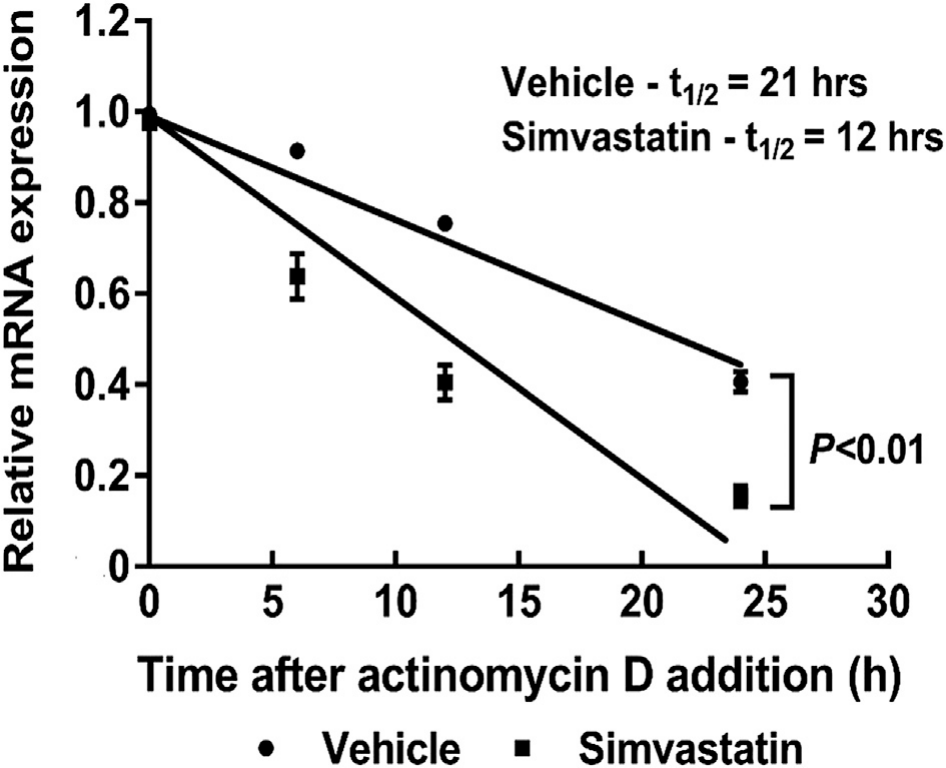
Simvastatin decreases PTTG1 mRNA stability. MDA-MB-231 cells were co-treated with simvastatin and actinomycin D (5 μg/ml) for 0, 3, 6, 12, 18, and 24 h. Cells were harvested for the extraction of total RNA and qRT-PCR was performed to determine the PTTG1 mRNA half-life. The data are representative of three independent experiments.

To further determine the molecular mechanism underlying inhibition of PTTG1 by simvastatin, we examined the PTTG1 mRNA stability by using the RNA polymerase inhibitor, actinomycin D. We found that the half-life of PTTG1 mRNA under basal cell culture conditions was 21 h ± 3 h (Figure 5), while treatment with simvastatin decreased PTTG1 mRNA half-life to 12 h ± 2 h. These data suggest that simvastatin inhibits PTTG1 expression primarily through decreasing PTTG1 mRNA stability in MDA-MB-231 breast cancer cells.

### Knockdown of Pituitary Tumor-Transforming Gene 1 Expression Attenuates MMP-2 and MMP-9 Activities

**FIGURE 6:**
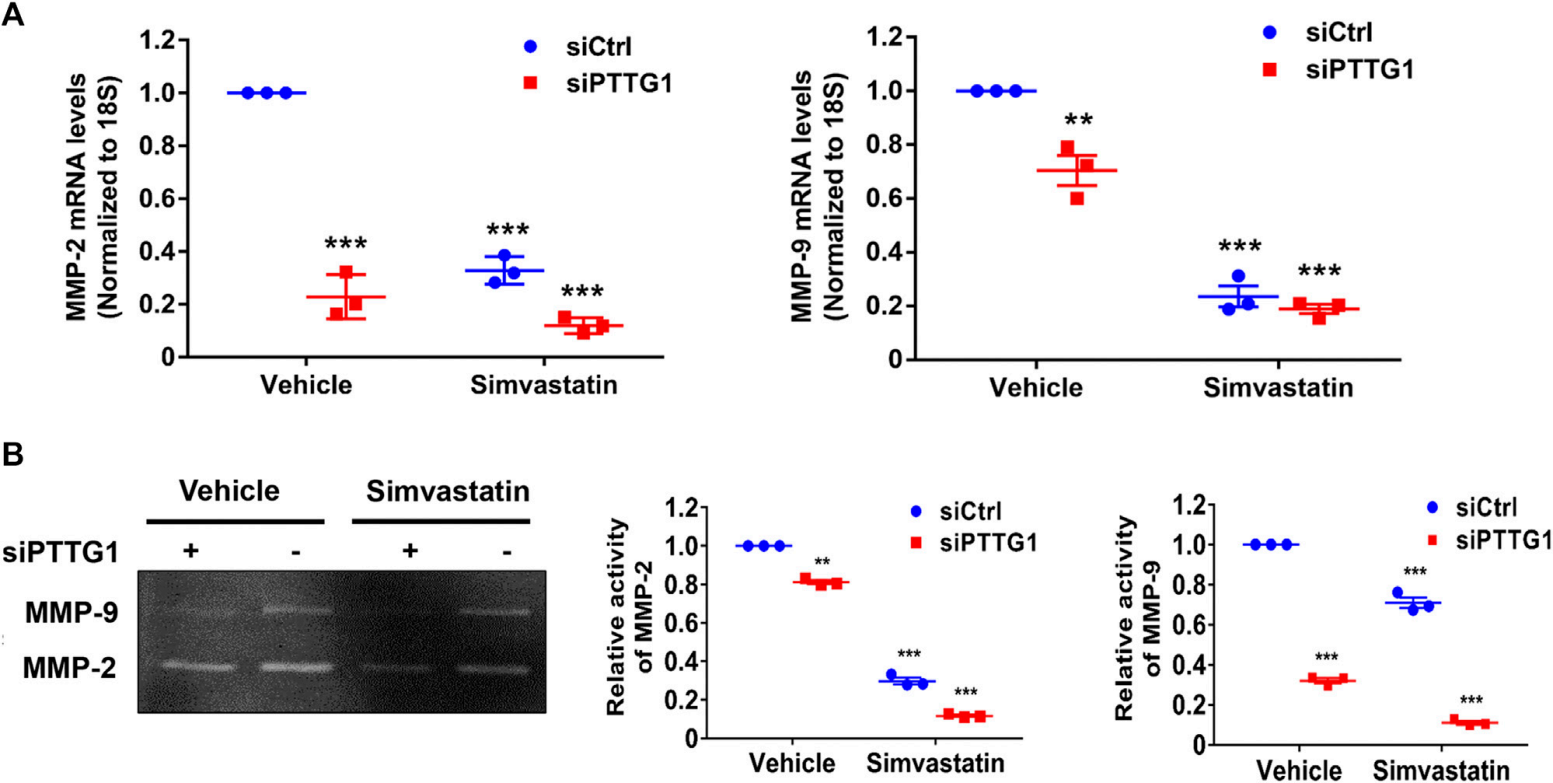
PTTG1 knockdown and simvastatin inhibit MMP-2 and MMP-9 activity. **(A)** MDA-MB-231 cells were transfected with control siRNA (siCtrl) and PTTG1 siRNA (siPTTG1). 48 h after transfection, cells were treated with either vehicle or simvastatin (5 μmol/L) for 24 h. The mRNA levels of MMP-9 and MMP-2 were then determined by qRT-PCR. Graphs depict relative levels of MMP-9 or MMP-2 mRNA for each sample, after normalization to 18s mRNA. Data are presented as the mean ± SEM for three separate experiments; **p < 0.01 vs. control group; ***p < 0.001 vs. control group. **(B)** MDA-MB-231 cells were transfected with control siRNA (siCtrl) and PTTG1 siRNA (siPTTG1). 48 h after transfection, cells were treated with either vehicle or simvastatin (5 μmol/L) for 24 h. Supernatants were subjected to gelatin zymography to examine the MMP-2 and MMP-9 activity. **p < 0.01 vs. vehicle; ***p < 0.001 vs. vehicle. The data are representative of three independent experiments.

To further determine the mechanism of PTTG1-mediated breast cancer cell invasion, we determined the effect of PTTG1 on the regulation of MMP-2 and MMP-9 expression and activity in MDA-MB-231 cells. As shown in Figure 6, we found that both siRNA-mediated knockdown of PTTG1 expression and simvastatin treatment significantly attenuated the expression of MMP-9 and MMP-2, as determined by qRT-PCR, and their enzymatic activities, as determined by gelatin zymography. Importantly, we found that PTTG1 knockdown demonstrated a more potent inhibition on MMP-2 than MMP-9 in MDA-MB-231 cells, which is consistent with previous observation demonstrating that MMP-2 is a direct target gene of PTTG1 (Hatcher et al., 2014).

### Simvastatin Inhibits the Expression of Pituitary Tumor-Transforming Gene 1 Downstream Target Genes

**FIGURE 7:**
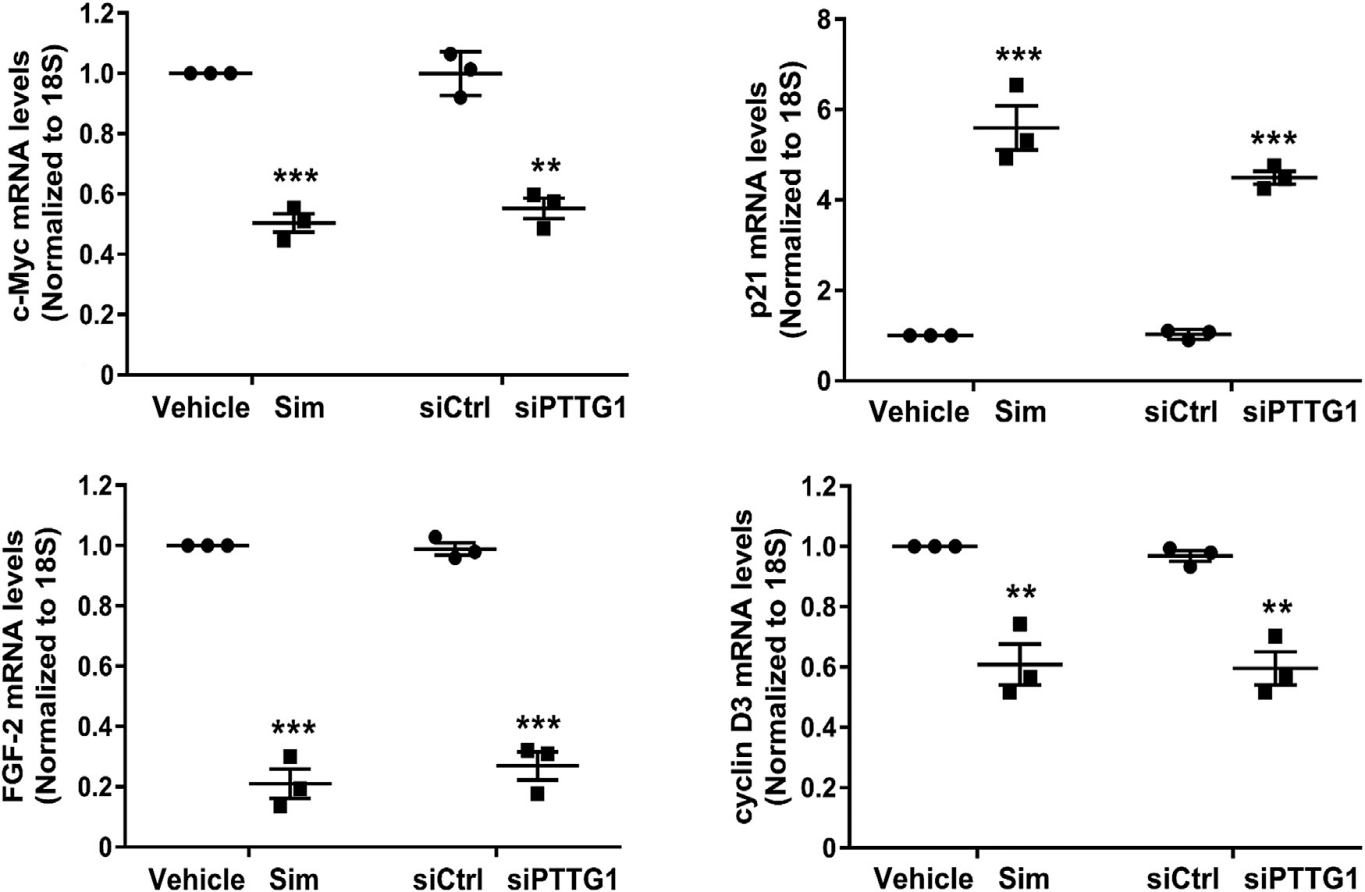
Simvastatin decreases the expression of PTTG1 downstream target genes. MDA-MB-231 cells were treated with 5 μmol/L simvastatin (Sim) for 24 h or transfected with control siRNA (siCtrl) or PTTG1 siRNA (siPTTG1) for 72 h. Cells were then harvested for the extraction of total RNA and qRT-PCR was performed to determine the expression of c-myc, FGF-2, p21, and cyclin D3. **p < 0.01 vs. vehicle or siCtrl; ***p < 0.001 vs. vehicle or siCtrl. The data are representative of three independent experiments.

PTTG1 has been shown to transcriptionally activate expression of a wide range of target genes, including c-Myc, FGF-2, cyclin D3, p21, and MMP-2, most of which are critically involved in cancer proliferation and metastasis (Espina et al., 2009; Tong and Eigler, 2009; Reeves et al., 2012). To examine whether simvastatin affects the expression of PTTG1 downstream target genes, qRT-PCR was performed to determine the expression of c-Myc, FGF-2, cyclin D3, and p21 in MDA-MB-231 cells treated with simvastatin for 24 h or transfected with PTTG1 siRNA or control siRNA for 72 h. As shown in Figure 7, both simvastatin treatment and PTTG1 knockdown significantly attenuated the expression of c-Myc, FGF-2, and cyclin D3 and increased p21 expression, implicating that simvastatin exerts anti-tumor effects through suppressing PTTG1 function in MDA-MB-231 cells.

### Involvement of Pituitary Tumor-Transforming Gene 1 in MDA-MB-231 Cell Invasion

**FIGURE 8:**
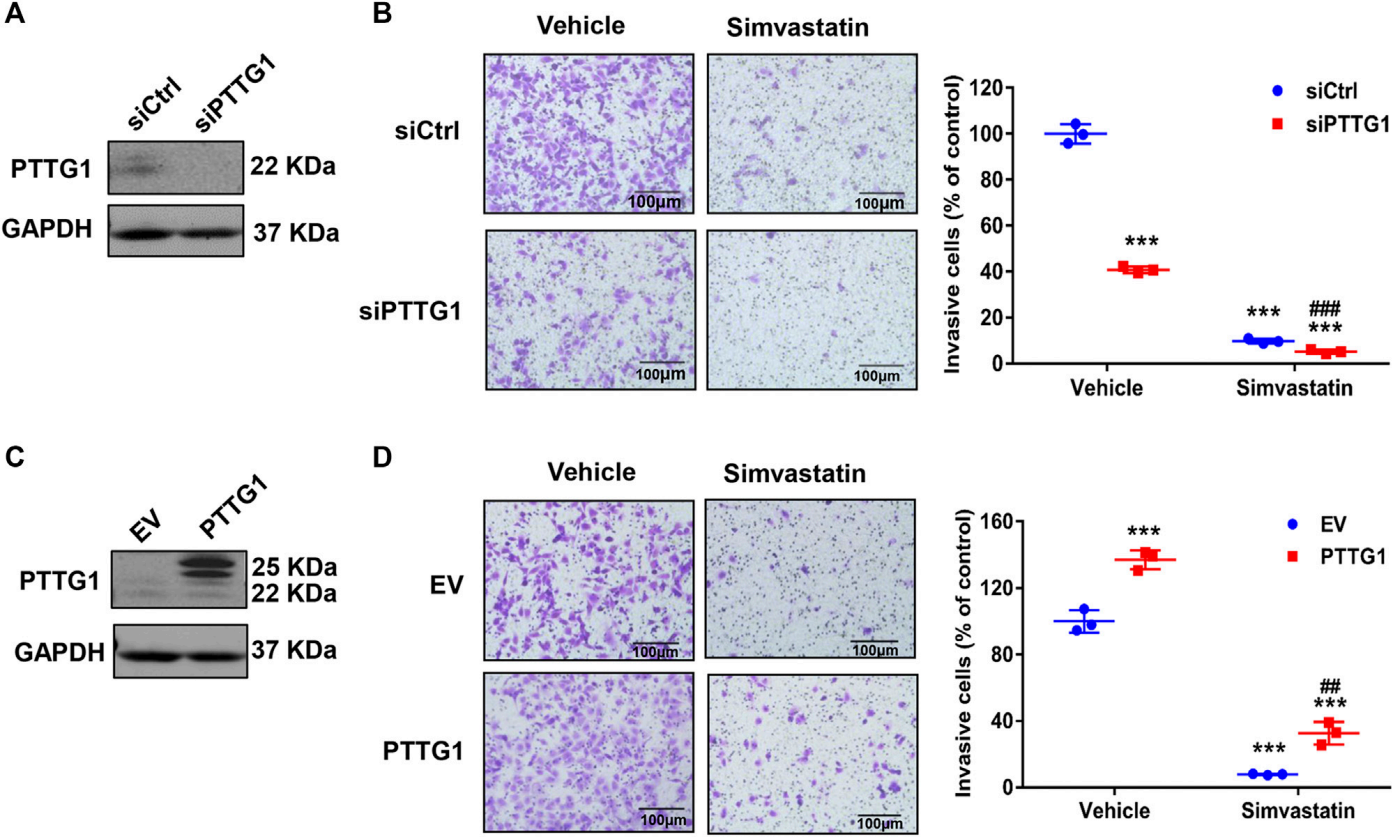
Regulation of MDA-MB-231 cell invasion by PTTG1 and simvastatin. **(A)** Protein expression of PTTG1 in MDA-MB-231 cells was decreased after transfected PTTG1 siRNA as determined by western blot. **(B)** Knockdown of PTTG1 inhibits MDA-MB-231 cell invasion. MDA-MB-231 cells were transfected with control siRNA (siCtrl) and PTTG1 siRNA (siPTTG1). 72 h after transfection, cell invasion was determined in the absence and presence of simvastatin (5 μmol/L) treatment for 24 h. ***p < 0.001 vs. control group, ^###^ *p* < 0.001 vs. simvastatin/siCtrl. **(C)** Increased expression of PTTG1 was determined in MDA-MB-231 cell transfected pCMV-Flag-PTTG1 or empty vector (EV) for 48 h. **(D)** Overexpression of PTTG1 increases basal invasion of MDA-MB-231 cells and partially reverses simvastatin-induced inhibition on MDA-MB-231 cell invasion. MDA-MB-231 cells were transfected with either empty vector (EV) or pCMV-Flag-PTTG1 expression plasmid. 48 h after transfection, cell invasion was determined in the absence and presence of simvastatin (5 μmol/L) for 24 h. ***p < 0.001 vs. control group, ^##^ *p* < 0.01 vs. simvastatin/EV. All experiments were carried out in triplicate. Data are presented as mean ± SEM.

To elucidate the role of PTTG1 in MDA-MB-231 cell invasion, we performed both gain- and loss-of-function studies. Matrigel invasion assay was carried out to determine breast cancer cell invasion. As shown in Figure 8, transfection of PTTG1 specific siRNA led to a marked inhibition of PTTG1 expression in MDA-MB-231 cells by approximately 90%. Accordingly, we found that in vehicle treated MDA-MB-231 cells, cell invasion, as determined by matrigel invasion assay, was significantly attenuated in PTTG1 siRNA transfected cells, as compared with control siRNA transfected cells. Moreover, simvastatin treatment substantially inhibited breast cell invasion in control siRNA transfected cells, and this inhibition was augmented in PTTG1 siRNA transfected cells. Meanwhile, we found that overexpression of PTTG1 enhanced cell invasion in vehicle-treated MDA-MB-231 cells, and partially reversed simvastatin-mediated inhibition on breast cancer cell invasion. Collectively, these results illustrated a critical role of PTTG1 in MDA-MB-231 breast cell invasion and suggested that inhibition of PTTG1 expression is contributed, at least in part, to the inhibition of breast cancer invasion by simvastatin treatment.

## Discussion

The human PTTG family has three isoform including PTTG1, PTTG2 and PTTG3. PTTG1 is a well characterized oncogene that is abundantly expressed in most invasive tumors and hematopoietic malignancies (Solbach et al., 2004; Yang et al., 2019), while the function of PTTG2 and PTTG3 remains relatively unknown. Consistent with previous reports (Solbach et al., 2004; Ogbagabriel et al., 2005; Yoon et al., 2012), we found that PTTG1 is highly expressed in malignant breast cancer cells. Furthermore, we found that lipophilic statins, including simvastatin, fluvastatin, mevastatin, and lovastatin, exhibited potent inhibitory effects on PTTG1 expression in metastatic MD-MBA-231 breast cancer cells, while hydrophilic pravastatin had no effect. Mechanistically, we showed that both knockdown of PTTG1 expression and simvastatin treatment potently attenuated breast cancer cell invasion, presumably through inhibition of the expression of PTTG1 downstream target genes, such as MMP-2, MMP9, c-myc, FGF-2, and cyclin D3. In MD-MBA-231 cells, simvastatin dose- and time-dependently inhibited PTTG1 expression through decreasing PTTG1 mRNA stability. In this regard, our study provide a strong mechanistic evidence supporting a beneficial effect of simvastatin in breast cancer management, as observed in recent several preclinical and clinical observations (Ahern et al., 2011; Liu et al., 2015; Van Wyhe et al., 2017).

Accumulating data suggest that statins have pleiotropic effects beyond lipid lowering (Liao, 2005; Van Wyhe et al., 2017). These effects include improvement of endothelial dysfunction, increasing nitric oxide bioavailability, antioxidant effects, and anti-inflammatory properties (Pinal-Fernandez et al., 2018). Recently, both preclinical and clinical studies have demonstrated beneficial effects of statins on breast cancer management (Van Wyhe et al., 2017; Beckwitt et al., 2018a; Li et al., 2019). Indeed, several *in vitro* and animal experiments have shown that statins exert positive anti-tumor effects by increasing breast cancer apoptosis (Åberg et al., 2008; Kamel et al., 2017), preventing carcinogenesis (Denoyelle et al., 2001), and inhibiting tumor growth (Wolfe et al., 2015; Beckwitt et al., 2018b). It should be noted that while most clinical studies showed positive effects of statins in management of breast cancer patients, while some clinical studies showed no or even opposite results (Borgquist et al., 2018). These discrepancies could be due to different genetic variants of breast cancers, use of different statins, and different durations of statin treatment. In the present study, we found that simvastatin markedly inhibited cell invasion in human breast cancer cell lines via down-regulating the expression of PTTG1, a key oncogenic gene involved in cancer invasion and metastasis (Grizzi et al., 2013). In this regard, our study is consistent with a recently published Danish study showing that simvastatin users exhibited a marked reduction in recurrence rates as comparing with non-statin users after 10 years of follow-up study of stage I–III breast cancer patients (Ahern et al., 2011).

PTTG1, also known as securin, is an important gene involved in many biological processes including inhibition of sister chromatid separation, DNA repair, organ development, metabolism, and angiogenesis (Vlotides et al., 2007; Grizzi et al., 2013; Hatcher et al., 2014). Increased expression of PTTG1 has been reported in proliferating cancer cells and various tumors including breast cancer, suggesting that PTTG may function in breast tumorigenesis (Yoon et al., 2012; Xiea and Wangb, 2016). For example, PTTG1 has been implicated in breast cancer development through regulation of the epithelial-mesenchymal transition (EMT) and functions of p27 and RhoA signaling (Quereda and Malumbres, 2009; Yoon et al., 2012; Lim et al., 2016). In addition, estrogen has been shown to promote breast cancer development through increasing PTTG1 expression (Chen et al., 2017; Meng et al., 2020). Although many studies have demonstrated the roles of overexpressing PTTG1 in breast cancer metastasis, the mechanisms underlying regulation of PTTG1 expression are still unclear. Particularly, identification of inhibitors of PTTG1 expression would provide novel therapeutic approaches for suppressing cancer metastasis. Herein, we for the first time demonstrated that in a time and dose-dependent manner, simvastatin significantly decreased both PTTG1 mRNA and protein expression levels in metastatic MDA-MB-231 cells. Accordingly, we showed that both simvastatin treatment and PTTG1 knockdown markedly attenuated MDA-MB-231 cell invasion possibly through suppressing MMP-2 and MMP-9 activities and expression of PTTG1 downstream target genes such as c-myc, FGF-2, and cyclin D3.

Mechanistically, we demonstrated that inhibition of PTTG1 expression by simvastatin occurs at posttranscriptional levels through decreasing PTTG1 mRNA stability. By inhibiting mevalonate synthesis, statins have been shown to inhibit the synthesis of isoprenoid intermediates thereby preventing isoprenylation of small GTPases, leading to the inhibition of these signaling molecules such as ROCKs (Laufs and Liao, 1998; Rikitake and Liao, 2005). In the present study, we found that inhibition of PTTG1 expression by simvastatin is reversed by GGPP, but not by FPP, suggesting the involvement of inhibiting protein geranylgeranylation, such as small GTPases, in this process. Indeed, treatment MDA-MB-231 cells with the ROCK inhibitor Y27632 led to a marked inhibition of PTTG1 to a similar extent as that seen in simvastatin treatment. Since small GTPases are critically involved in organizing the actin cytoskeleton (Rikitake and Liao, 2005), it would be interesting to determine whether disruption of actin cytoskeleton, which is a promising and intriguing anticancer strategy, inhibits PTTG1 expression in cancer cells. These studies are ongoing.

## Conclusion

Together, we for the first time identified statins as novel inhibitors of PTTG1 expression in breast cancer cells. Furthermore, our results provide mechanistic insights into how statins suppress breast cancer metastasis as observed in several preclinical and clinical studies. Furthermore, our results suggest that simvastatin may be more beneficial for breast cancer patients with high PTTG1 expression. In this regard, the current study provides an additional target for development of personalized medicine in management of breast cancer patients. Given a higher expression of PTTG1 in metastatic breast cancer, future studies examining the effect of statins on clinical outcomes of metastatic breast cancer patients is specifically warranted.

## Data Availability Statement

The raw data supporting the conclusions of this article will be made available by the authors, without undue reservation.

## Author Contributions

LY and JS conceived the idea for this study. LY, ZH, BY, LX, and JS interpreted the data and drafted the figures. LY, ZH, and JS performed the statistical tests. LY, ZH, BY, LX, and JS conducted the study. LY and JS wrote the original draft. All authors listed have made substantial, direct, and intellectual contribution to the work and approved it for publication.

## Funding

This work is supported by National Institutes of Health Grants (R01HL103869 and R01GM123047) and American Heart Association Established Investigator Award 16EIA27710023 to JS.

## Conflict of Interest

The authors declare that the research was conducted in the absence of any commercial or financial relationships that could be construed as a potential conflict of interest.

## Abbreviations

**Abbreviations:** FPP, farnesyl pyrophosphate; GGPP, geranylgeranyl pyrophosphate; HMG-CoA, 3-hydroxy-3-met hylglutaryl-coenzyme A; MMP, matrix metalloproteinase; PTTG1, pituitary tumor-transforming gene 1; qRT-PCR, quantitative real-time PCR; ROCK, Rho associated protein kinase; siRNA: small interference RNA.
